# La–Ni–Si: A Gold Mine with a Diamond

**DOI:** 10.1021/acs.inorgchem.4c03560

**Published:** 2024-11-18

**Authors:** Volodymyr Smetana, Davide Grilli, Vitalii Shtender, Marcella Pani, Pietro Manfrinetti, Anja-Verena Mudring

**Affiliations:** † intelligent Advanced Materials, Department of Biological and Chemical Engineering and iNANO, Aarhus University, 8000 Aarhus C, Denmark; ‡ Department of Materials and Environmental Chemistry, Stockholm University, Stockholm 10691, Sweden; § DCCI, Department of Chemistry and Industrial Chemistry, University of Genova, Genova 16146, Italy; ∇ Institute SPIN-CNR, Genova 16452, Italy; ∥ Department of Chemistry - Ångström Laboratory, Uppsala University, Uppsala 75121, Sweden; ⊥ Department of Physics, Umeå University, Linnaeus väg 24, 901 87 Umeå, Sweden

## Abstract

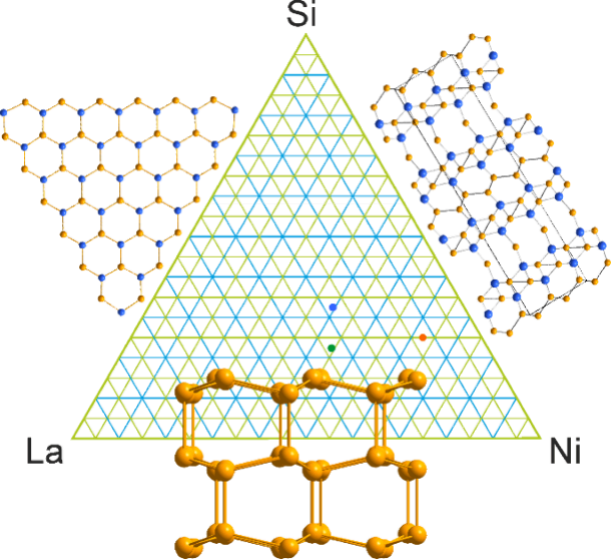

The La-poor part of the ternary La–Ni–Si system has been explored leading to the discovery and structural characterization of four new polar intermetallic compounds. LaNi_5_Si_2_ [BaAu_5_Ga_2_ type, *oP*64, space group *Pnma*, *a* = 7.8223(7) Å, *b* = 6.3894(6) Å, *c* = 17.843(2) Å, *V* = 891.8(2) Å^3^, *Z* = 8] features a diamond (lonsdaleite)-like homoatomic Ni framework and is the first Ni representative of a larger family of compounds typically formed by aurides. La_2_Ni_8_Si_3_ [Eu_2_Ni_8_Si_3_ type, *tP*52, *P*4_2_/*nmc*, *a* = 10.0278(3) Å, *c* = 7.5047(4) Å, *V* = 754.65(6) Å^3^, *Z* = 4] is characterized by homoatomic Ni_4_ tetrahedra and rectangles. LaNi_5_Si_3_ [SrNi_5_P_3_ type, *oS*36, *Cmcm*, *a* = 3.722(2) Å, *b* = 11.759(5) Å, *c* = 11.622(3) Å, *V* = 508.7(3) Å^3^, *Z* = 4] is governed by extensive heteroatomic bonding and characterized by homopolyhedral packing. La_3_Ni_4_Si_2_ [Ce_3_Ni_4_Si_2_ type, *mC*36, *C*2/*c*, *a* = 15.819(1) Å, *b* = 6.0068(5) Å, *c* = 7.4918(6) Å, β = 103.163(5)°, *V* = 693.17(10) Å^3^, *Z* = 4] is a new member of homologous series that includes La_3_Ni_3_Si_2_ and La_3_Ni_3.5_Si_2_. We conclude that the similar radii and electronegativities of Ni and Si are the reason for the incredible diversity of compositions and structures in this system.

## Abstract 2

The La−Ni−Si system has been explored in the Ni-richer corner reaching 33 compounds in total, including the first for a Ni homoatomic lonsdaleite framework.

## Introduction

Although formally not a metal, silicon exhibits a crystal chemistry that is frequently identical or very close to pure intermetallics.^1,2^ Metal silicides occur for a vast abundance of compositions and feature a large variety of structural motifs.^3^ Therefore, intermetallic silicides form a class of compounds that links true intermetallics and ionic compounds. This is also reflected in the materials properties and silicides have found broad applications in high-temperature coatings,^4,5^ microelectronics,^6,7^ catalysis,^8^ etc. Ternary silicides, particularly those with rare-earth and/or transition metals are famous for their giant magnetocaloric effect,^9,10^ superconductivity^11,12^ or hydrogen sorption properties.^13^

As Si is, on the one hand, close in size^14^ and electronegativity^15^ to other metalloids as well as some nonmetals, and on the other, late 3*d* metals, a wide range of compositions can be realized is such systems, which gives plenty of possibilities for property tuning. Being a very abundant element in nature, silicon allows for production scaling circumventing any supply interruptions. A particularly rich chemistry can be observed for ternary silicides with transition metals and lanthanides.^3^ The respective ternary systems frequently feature good miscibility and, consequently, good variability of compositions and high structural diversity.

The La–Ni–Si system recently attracted our attention due to the enormous richness of the reported compositions with all the new findings being stunningly detected in compositionally small narrow regions frequently forming closely related series.^16−18^ Efforts to investigate the phase equilibria in this system in detail point to an even larger number of hidden islands of compounds. Interestingly, a preprint on phase equilibria at 1070 K^19^ misses all these recent findings, while phase equilibria reported at 673 K^20^ are limited to just 14 ternary compounds. Although some phases may undergo an eutectoid transformation, it appears unrealistic that half of the compounds are missing at lower temperatures (especially in the light of large number of samples claimed to be prepared). Consequently, both works can hardly be considered complete and refer to outdated/incomplete results, and the phase equilibria there must be revisited, if not reinvestigated from scratch. In this work, we report the synthesis and structural characterization of four new ternary silicides: LaNi_5_Si_2_, La_2_Ni_8_Si_3_, LaNi_5_Si_3_ and La_3_Ni_4_Si_2_.

## Experimental Section

### Synthesis

All alloys were prepared starting from the same pure elements: La pieces (99.9 wt %), Ni slugs (99.99 wt %) and Si grains (99.999 wt %). Samples with different compositions (La_9.5_Ni_57.2_Si_33.3_, La_17.8_Ni_54.6_Si_27.6_, La_33.34_Ni_44.44_Si_22.22_, La_12.5_Ni_58.75_Si_28.75_) and a total mass of about 1 g each were arc melted in high-purity argon atmosphere using Ti ingot as a getter. The buttons were remelted at least twice after turning them upside-down to ensure good homogenization. Weight losses after arc melting never exceeded 0.5 wt %. After melting, the as-cast samples were placed in Ta foil, closed in evacuated fused silica tubes and annealed at 1273 K for 7–14 days. The La_3_Ni_4_Si_2_ sample was annealed at 873 K for 1 month. At the end of the heat treatment, samples were cooled to room temperature by switching off the furnace. Single crystals of the new ternary compound were isolated from the crushed samples. The samples were stable in air for at least months in any form.

### Single-Crystal and Powder X-ray Diffraction

Powder patterns were collected using a Panalytical powder diffractometer (Bragg–Brentano geometry, Ni-filtered Cu Kα radiation) in the 10–90° 2ϑ range, with 0.02° 2ϑ step and counting times of 15–20 s per step. Phase analyses were performed using the WinXPow 3.1 program (STOE & Cie GmbH). The FullProf program package was applied for Rietveld analysis of the collected data sets.^21^ Selected powder pattern Rietveld refinements have been provided in Supporting Information revealing the complexity of the phase space with multiphase products (Figures S1–S4).

Single-crystal X-ray diffraction (XRD) measurements were performed at room temperature on a Bruker D8 Venture diffractometer operating at 50 kV and 1.4 mA equipped with a Photon 3 CMOS detector, a flat graphite monochromator, and a Mo Kα IμS 3.0 microfocus source (λ = 0.71073 Å). The raw frame data were collected and handled using the Bruker APEX3 software package (Bruker AXS, 2015). Absorption correction has been performed using the multiscan method (SADABS).^22,23^ Initial models of the crystal structures were obtained with the program SHELXT-2014^24^ and refined with SHELXL-2014^25^ within the APEX3 software package. Anisotropic displacement parameters have been refined for all atoms. Reflection intensities and symmetries were carefully re-examined with the aid of the PLATON program to exclude any missing higher symmetry.^26−29^ The program DIAMOND was used for drawing and analyzing the crystal structure.^30^

### Electronic Structure Calculations

Tight binding electronic structure calculations for idealized LaNi_5_Si_2_ were performed according to the linear muffin-tin-orbital (LMTO) method in the atomic sphere approximation (ASA).^31,32^ The problem of occupational disorder has been treated by assigning fully occupied Ni and Si positions, respectively, for both Ni/Si positions. The only drawback of this model is the appearance of short Si–Si bonds, which do not exist in the real structure and will be discussed separately (see below). The radii of the Wigner-Seitz spheres were assigned automatically to guarantee the best match between the overlapping and full potentials.^33^ They were determined to be 2.22 and 2.06 Å for La, 1.33, 1.31, 1.36, 1.31, 1.32, and 1.30 Å for Ni and 1.37, 1.39, 1.38, and 1.38 Å for Si. No empty spheres were needed to fully occupy the space. The basis sets were 6*s*/(6*p*)/5*d*/4*f* for La, 4*s*/4*p*/3*d* for Ni and 3*s*/3*p*/(3*d*) for Si with orbitals in parentheses downfolded.^34^ The convergence criterion was set to 10^–5^ eV. The band structure was sampled for 196 k-points in the irreducible wedges of the Brillouin zone. Crystal orbital Hamilton populations (COHPs) as well as their weighted sums (ICOHPs)^35^ have been calculated and analyzed to provide insights into the bonding situations in the crystal structure.

## Results and Discussion

Our previous screening of the La–Ni–Si system revealed that, despite intensive explorations, such systems may still contain unexplored or overlooked areas with plenty of unknown compounds.^16,17^ Indeed, research in similar systems has intensified recently confirming our expectations. For instance, the isothermal section of the Ce–Ni–Si system was constructed just in 2016,^36^ however, a very fresh investigation from 2024 (just a few months ago), revealed additional compounds following isocompositional findings in the La–Ni–Si system.^37^ Although obtained in the same system, some compounds have different stability ranges that may frequently not overlap.

LaNi_5_Si_2_ crystallizes in the orthorhombic space group *Pnma* (*a* = 7.8223(7), *b* = 6.3894(6), *c* = 17.843(2) Å, *V* = 891.8(2) Å^3^, *Z* = 8) and belongs to the larger family of compounds formed by a combination of group II–III metal/transition metal/*p*-element and which is represented mostly with Au as the transition metal (see Table 1). A prominent feature of the family is the formation of a transition metal homoatomic framework with the structure of lonsdaleite, which is sometimes also addressed as hexagonal diamond.^38^ It is interesting to note that such a framework has never been observed for any pure metal, while the reported structure of lonsdaleite itself is still under discussion and may not exist independently.^39,40^ A quick inspection of the phase space around the discovered compositions showed the existence of isostructural CeNi_4.95_Si_2.05(1)_ (*a* = 7.774(2), *b* = 6.390(2), *c* = 17.539(5) Å, *V* = 871.2(4) Å^3^), while homologous honeycomb compounds specifically in the La–Ni–Si system are not excluded although have not yet been confirmed.

**Table 1:** Overview of Representative Compounds Featuring a Metal Lonsdaleite Framework

	M/Ae^a^	space group	ST	*a* (Å)	*b* (Å)	*c* (Å)	β (deg)	refs
BaAg_4.9_Al_2.1_	7	*P*62*m*	own	8.913		7.278		(41)
SrAu_4_Ga_3_	7	*P*62*m*	BaAg_4.9_Al_2.1_	8.609		7.204		(42)
SrAu_5_Al_2_	7	*Pnma*	own	8.942	7.232	9.918		(43)
BaAu_5_Ga_2_	7	*Pnma*	own	8.855	7.188	20.37		(44)
LaNi_5_Si_2_	7	*Pnma*	BaAu_5_Ga_2_	7.8223(7)	6.3894(6)	17.843(2)		
Ba_1.04_Au_4.5_Ga_2.4_	6.6	*P*62*m*	own	8.789		7.212		(44)
Eu_1.1_Au_4.4_Ga_2.2_	6	*P*62*m*	own	8.543		7.249		(44)
Ae_2_Au_6_T_3_	4.5	*R*3*c*	own	∼8.72		∼21.3		(45−49)
Ae = Sr, Ba, or Eu; T = Zn, Cd, Al, Ga, In, or Sn								
Sr_2_Au_7_T_2_	4.5	*C*2/*c*	own	∼15.0	∼8.56	∼8.67	∼124	(43), (45)
T = Zn, Al, or Ga								
Ca_4_Au_10_Zn_3_	3.25	*C*2/*c*	own	15.98	8.197	11.88	120.9	(50)
Cu_2.88_As	–	*P*3*c*1	own	7.110		21.879		(51)

a M/Ae = anion/cation ratio.

**Figure 1:**
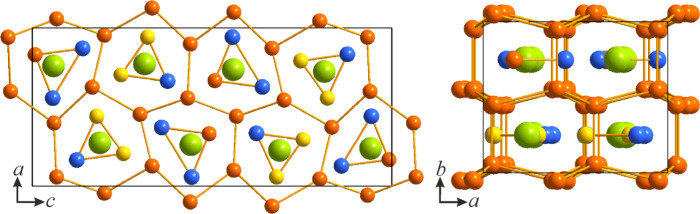
Projections of the crystal structure of LaNi_5_Si_2_ along the *b* (left) and *c* (right) crystallographic axes. Honeycombs are alternatively filled with La or Ni/Si triangles. La atoms are colored green, Ni atoms orange, Si atoms blue, and mixed Ni/Si positions yellow.

As LaNi_5_Si_2_ is isostructural to BaAu_5_Ga_2_,^44^ it will primarily be compared to the latter. In LaNi_5_Si_2_, the diamond-type network is formed of Ni atoms, while the honeycomb cavities are filled with single La atoms or NiSi_2_ triangles (Figure 1). Although the compound appears to be a Daltonide, a significant amount of occupational disorder involving two Ni/Si positions is observed. These positions show preferred occupation by Ni and Si, respectively, while the ratio is well counterbalanced practically approaching stoichiometric composition. Identical behavior was observed in the prototype compound as well. In LaNi_5_Si_2_, there are two types of Ni/Si triangles; those involving disordered Ni/Si sites are more regular (*d*_Ni–Ni_ and *d*_Ni–Si_ are in the range 2.283–2.397(6) Å), while those with fully occupied pure Ni and Si positions are significantly elongated showing visible separation of the Si vertices (*d*_Ni–Si_ = 2.333–2.385(6) Å and *d*_Si–Si_ = 2.889(7) Å). This distortion is more substantial than in the parent Au compound and may be due to competing strong Ni–Si interactions within the triangles and between them and the framework. Ni/Si triangles and La alternate within the framework honeycombs.

It is worth noting that the formation of the diamond framework was attributed to the relativistic effects of Au and to the presence of corresponding strong Au–Au interactions helping to stabilize the construct.^43^ That consideration was in line with the experimental discoveries that all observed compounds featuring a lonsdaleite substructure were observed for Au compounds, with just one Ag representative as an exclusion (Table 1). Although Au and Ag bear many similarities, their intermetallic structural chemistry usually shows substantial differences, particularly in complex intermetallics.^52^ In this light, the observation of another isostructural compound with Ni is astonishing, although corresponding 2D honeycomb constructs with Ni are known.^53,54^ As a separate branch, we shall also mention the LaF_3_ structure type^55^ where lonsdaleite motifs are formed with uniform filling of the honeycombs although without dominating homoatomic bonding. In this context, the Cu_3_M (M = P, As) binaries^51^ which crystallize with the anti-LaF_3_ type of structure are interesting as they offer additional possibilities for discovery of lonsdaleite frameworks with other transition metals. In this respect, we analyzed geometric and electronic patterns throughout the entire series.

From the geometric point of view, it sounds logical that smaller La (covalent radius = 2.07 Å) forms the structure with smaller Ni and Si (1.24 and 1.11 Å),^14^ while bulkier Ba (2.15 Å) was observed with bigger Au and Ga (1.36 and 1.22 Å). Unfortunately, we cannot extend this analysis for the entire series as structural distortions affect geometric criteria, and no other Ni/Si structures have been detected yet. For instance, the smallest Ca (1.76 Å) forms a singularity in the family, belonging to a monoclinic structure with no reported isostructural compounds yet,^50^ while the biggest Ag (1.45 Å) is known only in combination with the biggest Ba (2.15 Å).^41^ Even the isocompositional compound SrAu_5_Al_2_ with the slightly smaller Sr (1.95 Å) revealed some geometric changes (Table 1) to stabilize the lonsdaleite framework. Although it is rather hard to predict new element combinations, more extensive synthetic explorations may help to complement the database of lonsdaleite frameworks in intermetallics and perform a comprehensive analysis.

From the electronic side, since the framework is formed of electron-poorer Ni, both the cation (La) and the *p* element (Si) are electron-richer, pointing to an electronic origin of the structure stabilization. In fact, Au compounds are electron-richer having *vec* (valence electron count) 13 for the isocompositional compounds (BaAu_5_Ga_2_ and SrAu_5_Al_2_) compared to 11 in LaNi_5_Si_2_. To further elucidate this aspect, we continued with the inspection of the electronic structure and bonding picture along the series.

**Figure 2:**
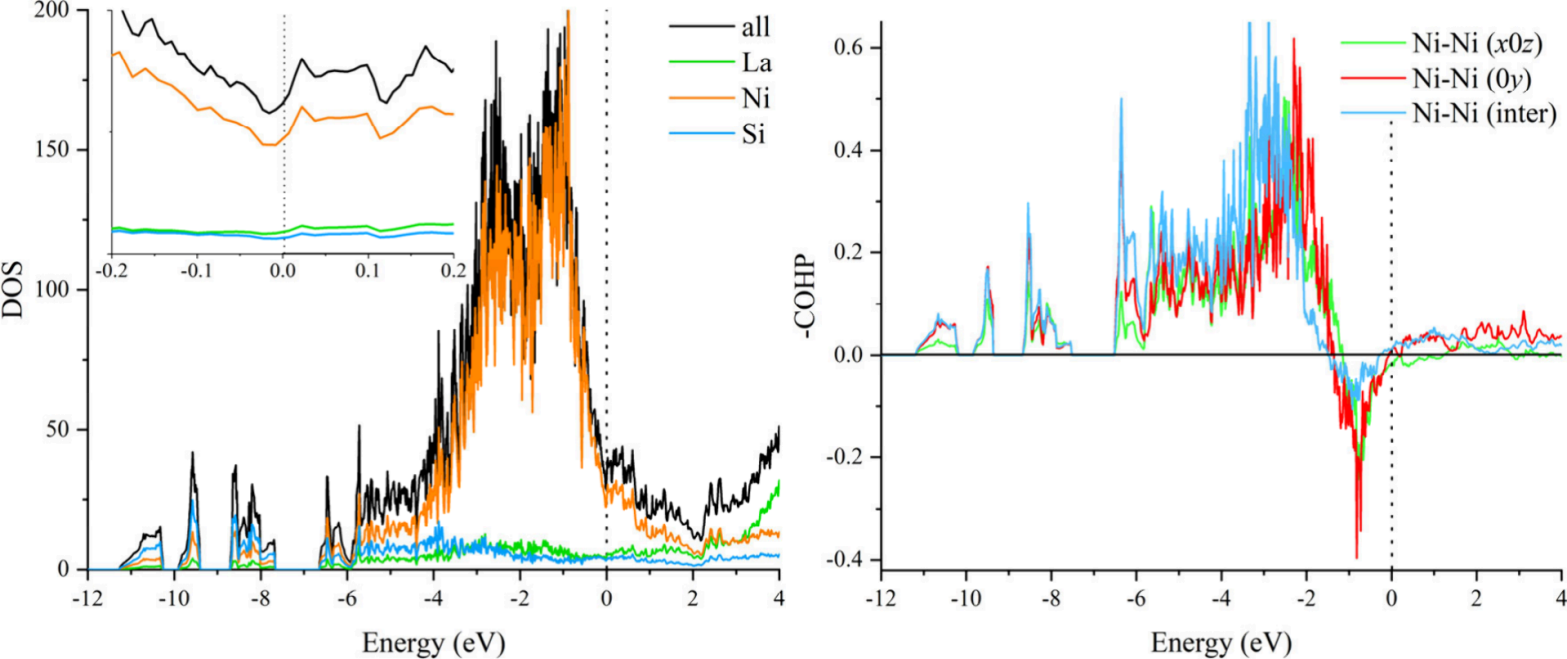
Density of states (left) and crystal orbital Hamilton populations (right) for LaNi_5_Si_2_. The Fermi levels are marked with dotted lines.

The electronic density of states (DOS) is characterized by broad overlaps across the entire energy range and a small local minimum at the Fermi energy, *E*_F_, being typical for intermetallic compounds (Figure 2 and S5). The area between −6 and +1 eV is dominated by Ni-*d* states. La-*f* states can be observed at energies ≥3 eV. A distinct pseudogap could only be observed at around 2 eV above the Fermi level and can only serve as an indication of the preference of the structure type to accommodate more electrons following the experimental observations. On the other hand, Ni-*d* states are located directly at the Fermi level pointing toward the possibility of their inclusion in the *vec* schemes to compensate for the electron deficiency. The band structure of LaNi_5_Si_2_ (Figure S6) indicates some electronic instability at the Fermi level, particularly around Y and G points, that may be due to possible magnetic ordering of Ni atoms or superconductivity. Performing a spin-polarized calculation for deeper analysis is difficult because of the structural complexity. However, it has to be noted that, Ni mostly does not order magnetically in similar compounds.^56^ Superconductivity has though been observed at 1.8 K in another disordered La–Ni silicide La_5_Ni_1.75_Si_3_.^57^

A comprehensive analysis of the bonding in LaNi_5_Si_2_ has been performed using the crystal orbital Hamilton populations COHP scheme with particular focus on bonding patterns within the homoatomic Ni diamond framework, see Figure 2. While the Ni–Ni interactions are all bonding up to about −1 eV, they turn strongly antibonding at this point up to the Fermi level. Such a situation has been observed in intermetallics for other homoatomic transition metal interactions, like Au, Pd or Pt and is commonly associated with *d*^10^-*d*^10^ repulsion.^58−64^ A subtle difference can be observed in the Ni–Ni interactions along different crystallographic directions. Ni–Ni interactions in the six-membered Ni rings with boat-conformation running parallel to the *b* axis, normal to the NiSi_2_ triangular plane, are bonding at *E*_F_ and stay bonding well above it. The same was observed for the Ni–Ni interactions between the lonsdaleite framework and the NiSi_2_ triangles, while Ni–Ni contacts within the condensed network of six-membered Ni rings with chair-conformation in the *ac* plane are antibonding up to 1 eV above the Fermi level. A similar bonding behavior has also been observed in the gold prototype.^44^ However, relativistic effects of gold may play a significant role in softening those antibonding interactions allowing for a large diversity of the homoatomic motifs.^65^ Thus, homoatomic transition metal framework is not stable on its own, justifying its nonexistence with pure metals, but needs supporting interactions for stabilization.

It is not unexpected that heteroatomic Ni–Si bonds provide the largest contribution to the total bonding (Table 2 and S10), particularly those between the triangles and the Ni framework. Although numerous, the contributions from the Ni–Ni pairs are significantly smaller yet comparable to Au–Au’s contribution in the related Au lonsdaleite structures.^46^ Interestingly, the number and contributions from the framework Ni–Ni pairs and supporting Ni–Ni interactions involving NiSi_2_ triangles are practically identical. Populations involving the formal cation La, result in 14% of the total. This number is high with respect to alkali and alkaline-earth interactions,^44,66^ but indicates a somewhat lower involvement of La in the bonding with Ni compared to heavier transition metals.^58,63^ Somewhat higher numbers for the Si–Si interactions are due to the model used for the calculation and, in part, represent Ni–Si interactions. True Si–Si (2.878 Å) as well as La–La contacts represent a negligible component of the interactions in LaNi_5_Si_2_.

**Table 2:** Bond Lengths, −ICOHP Values, and Bonding Contributions in LaNi_5_Si_2_

bond type	length (Å)	–ICOHP (eV/average bond)	no. per cell	–ICOHP (eV/cell)	contribution (%)
Ni–Ni (0*y*)	2.496–2.593	0.97	16	15.5	3.8
Ni–Ni (*x*0*z*)	2.583–2.805	0.85	40	34.1	8.3
Ni–Ni (inter)	2.432–2.522	1.29	48	61.8	15.1
					27.2
Ni–Si (triangular)	2.272–2.375	2.02	28	56.6	13.8
Ni–Si (inter)	2.301–2.497	1.88	92	174	42.4
					56.2
La–Ni	2.918–3.443	0.34	120	41.3	10.1
La–Si	3.168–3.611	0.28	56	15.8	3.8
Si–Si	2.385–2.878	1.35	8	10.7	2.6
La–La	4.412–4.497	0.06	8	0.45	0.1

**Figure 3:**
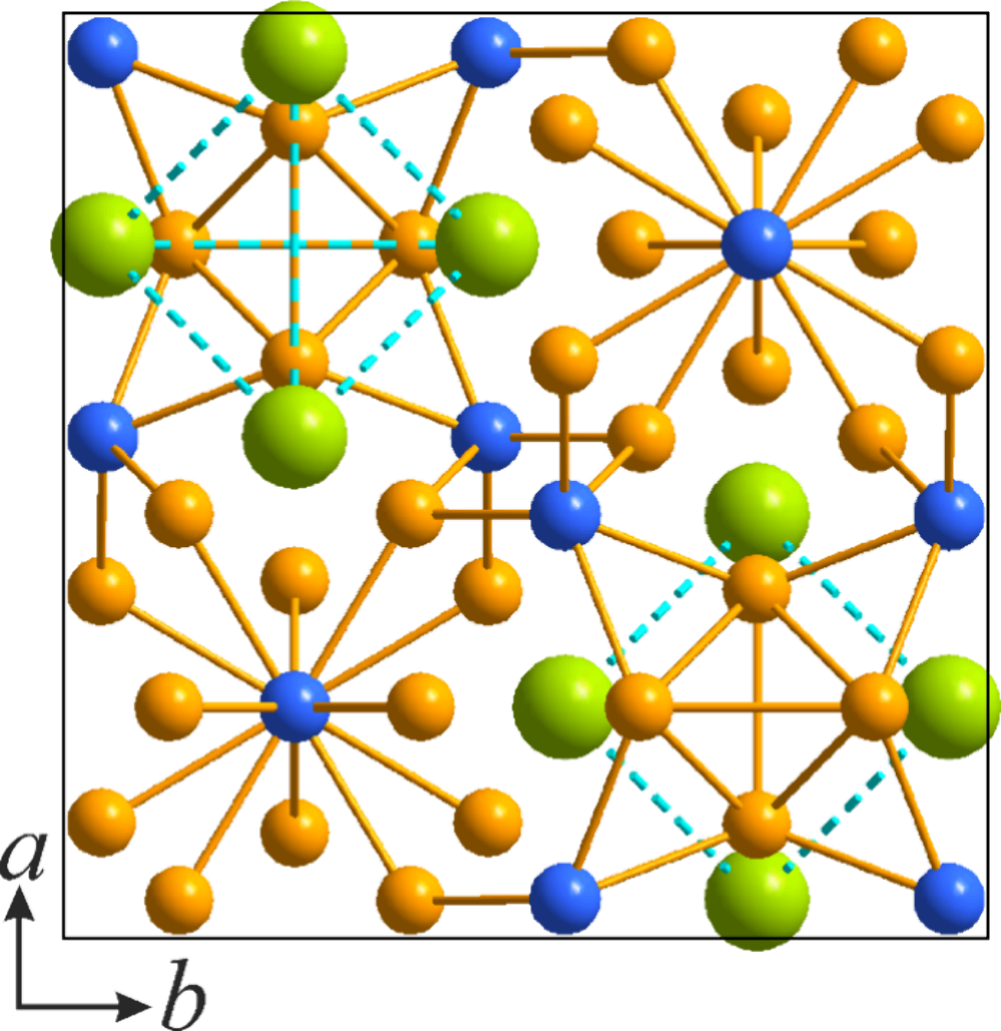
Projection of the crystal structure of La_2_Ni_8_Si_3_ along the crystallographic *c* axis. La atoms are colored green, Ni atoms orange, and Si atoms blue.

La_2_Ni_8_Si_3_ crystallizes in the tetragonal space group *P*4_2_/*nmc* (*a* = 10.0278(3), *c* = 7.5047(4) Å, *V* = 754.65(6) Å^3^, *Z* = 4) and crystallizes with the Eu_2_Ni_8_Si_3_ type^67^ and its antitype analogue Sr_2_Pt_3_Al_8_.^68^ The crystal structure can be described based on two sets of tetrahedral motifs alternating along three crystallographic axes. In the first one, La_4_ tetrahedra alternate along the *c* axis with Ni_4_ tetrahedra, each surrounded by a Si_4_ square (Figure 3). The second set is purely anionic and consists of edge-sharing SiNi_4_ tetrahedra surrounded by Ni_4_ rectangles. The connectivity between both sets is established solely via Ni–Si contacts between the Si_4_ squares and the Ni_4_ rectangles surrounding the inner tetrahedra.

The coordination environment of two Ni positions is trigonal antiprismatic (Ni@Ni_6_) with a different degree of capping. One of them is capped equatorially by two Si and three La atoms, while the other is by a Si_4_ tetrahedron and three additional La atom. The last Ni is coordinated by five Ni, three Si and three La positions in the form of a distorted tricapped (Ni+Si+La) tetragonal prism (Ni@Ni_4_Si_2_La_2_). Si coordination environments are best described as tetracapped (2 Ni + 2 La) Si@Ni_6_ trigonal prism and a slightly distorted Frank-Kasper polyhedron with CN = 12 (8 Ni + 4 La). Due to the segregation of the La atoms forming tetrahedra, the coordination environment of the latter is highly irregular having CN = 17 (La@Ni_9_Si_5_La_3_). It is interesting to note that despite higher symmetry the distribution of the Ni–Si and Ni–Ni distances is quite broad -2.272–2.608(2) and 2.393–2.731(1) Å. Both lower edges of the ranges start slightly below the sum of the corresponding covalent radii. Similarly to La_3_Ni_4_Si_2_, no Si–Si bonds could be observed here. Not surprisingly (as the compositions are just 3 at. % away from each other) the entire bonding spectrum is equally represented by the cation–anion (La–Ni(Si)) contacts − 72 + 40, Ni–Ni – 88, and Ni–Si bonds − 92 showing very close proportions to LaNi_5_Si_2_ (see Table S7) and correspondingly similar ICOHP contributions (Table S11).

LaNi_5_Si_3_ crystallizes in the orthorhombic space group *Cmcm* (*a* = 3.722(2), *b* = 11.759(5), *c* = 11.622(3) Å, *V* = 508.7(3) Å^3^, Z = 4) and belongs to the SrNi_5_P_3_ structure type being the first silicide representative of the latter. Practically all compounds crystallizing in that type have also been reported with Ni and large formal cations, i.e. Sr, Eu and La^69−72^ suggesting the high importance of the geometric factor for this type of structure.

**Figure 4:**
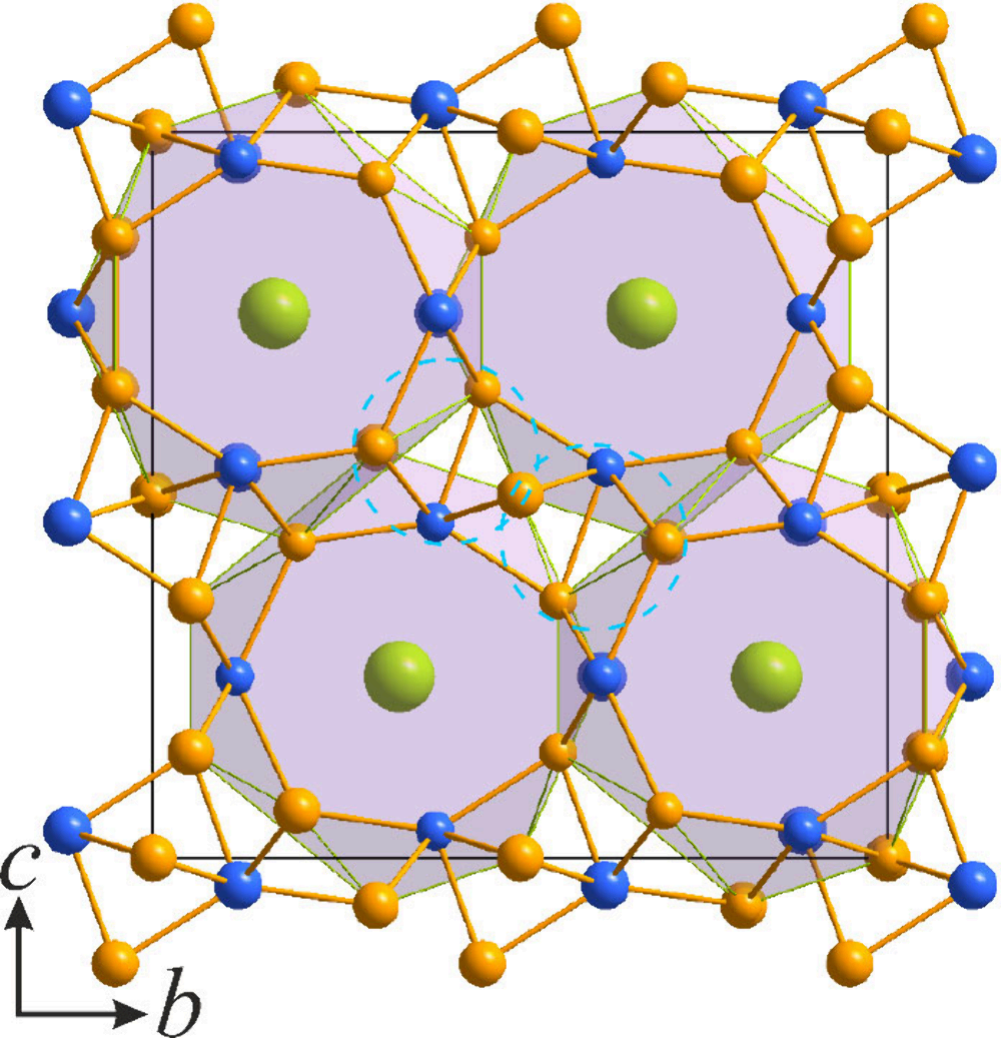
Projection of the crystal structure of LaNi_5_Si_3_ on the *bc* plane. La atoms are colored green, Ni atoms orange, and Si atoms blue.

The crystal structure of LaNi_5_Si_3_ consists of a set of straight tunnels extending along the *a* axis (Figure 4). The tunnels are formed by stacking La@Ni_14_Si_7_ polyhedra with two large open faces. Such polyhedra consist of three parallel planar 7–7–7 membered rings. It is worth noting that despite the high diversity this population of rings has not been observed in the intermetallics with larger Au.^65^ The neighboring tunnels share common triangular faces but such stacking leads to solely anionic chains between each three of them in the form of stacking edge-sharing tetrahedra. The latter form dumbbells via common vertices (Figure 4, dashed circles).

All Ni positions in the structure exhibit a distorted equatorially tetracapped tetragonal prismatic or highly distorted icosahedral environment (CN = 12) – Ni@Ni_7_Si_2_La_3_, Ni@Ni_5_Si_4_La_3_ and Ni@Ni_6_Si_4_La_2_. Si positions have coordination numbers 9 and tricapped trigonal prismatic coordination – Si@Ni_6_La_3_ and Si@Ni_7_La_2_. All polyhedra for this and the following structures have been illustrated in Figure S7. Ni–Si and Ni–Ni contacts in the structure are in relatively narrow ranges of 2.2331–2.4045(9) and 2.4291–2.7160(7) Å. Practically all Ni–Si contacts are below the sum of the corresponding covalent radii suggesting strong interactions. Interestingly, Si–Si contacts of 2.865(1) Å are present being comparable to those found in LaNi_5_Si_2_. Of course, such distance is far away from any bonding interaction highlighting the tendency of Si atoms to avoid any direct interaction. A somewhat higher Si proportion in the formula unit is reflected in the number of Ni–Si contacts in the structure −68 as compared to Ni–Ni–48 or La–Ni + La–Si–40 + 28, respectively (Table S8). This points to optimization of the heteroatomic bonding in contrast to Ni–Ni clustering in two previous structures.

**Figure 5:**
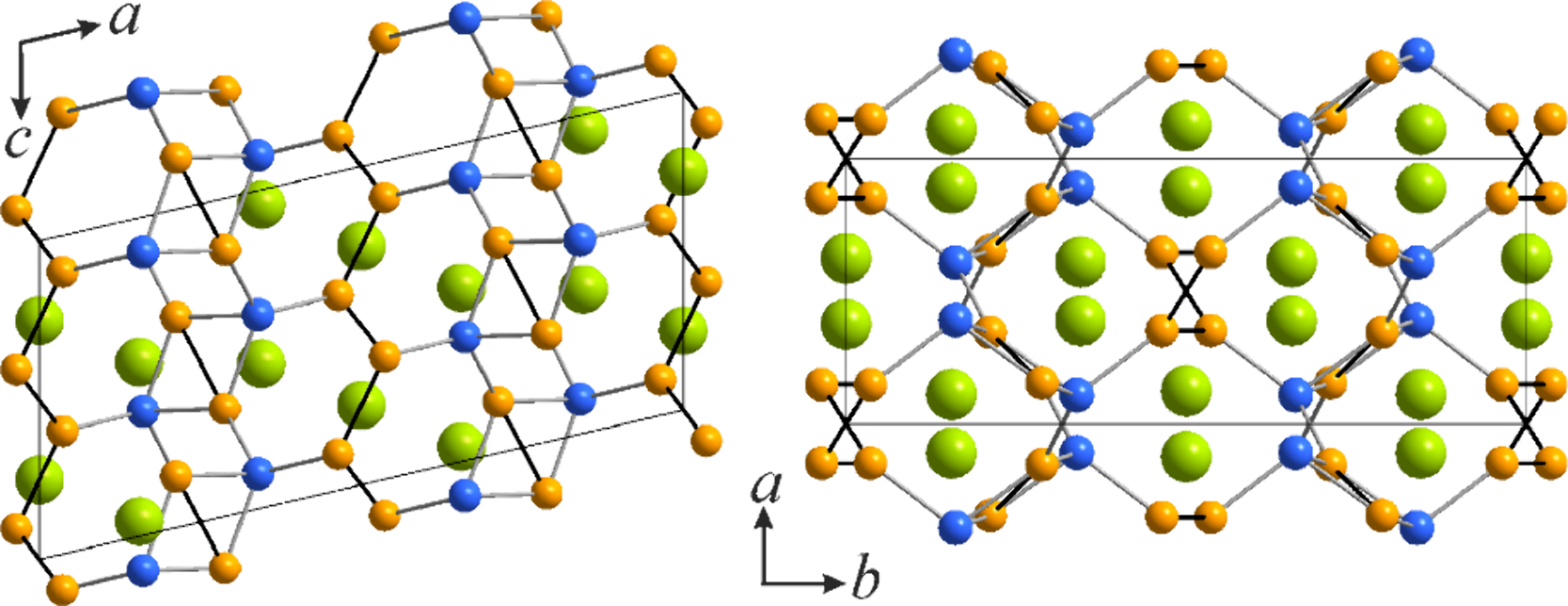
Projection of the crystal structure of La_3_Ni_4_Si_2_ on the *ac* and *ab* planes, respectively. La atoms are colored green, Ni atoms orange, and Si atoms blue.

La_3_Ni_4_Si_2_ was not detected in the samples that were annealed at 1270 or even 1070 K while good quality crystals were observed after slow cooling and prolonged annealing at 870 K. This compound crystallizes in the Ce_3_Ni_4_Si_2_ type of structure^37^ (SG *C*2/*c*, *a* = 15.819(1), *b* = 6.0068(5), *c* = 7.4918(6) Å, β = 103.163(5)°, *V* = 693.17(10) Å^3^, *Z* = 4). Its crystal structure is best represented in terms of polyanionic framework with encapsulated formal cations, *i*.*e*. La atoms (Figure 5). Ni and Si atoms form tunnels along the *c* axis, while La atoms are arranged in zigzag chains inside. From this point of view, the structure is quite uniform although the Ni/Si ratio may vary within the tunnels around two different La sites. Due to the higher electronegativity of La, it is less of an electron donor in contrast to alkali metal and participates more significantly in covalent bonding. Therefore, the polyanionic tunnel structure contains more open faces in contrast to similar compounds with active metals, *e*.*g*. *A*Au_3_Ga_2_ (*A* = K–Cs).^59,60^

**Figure 6:**
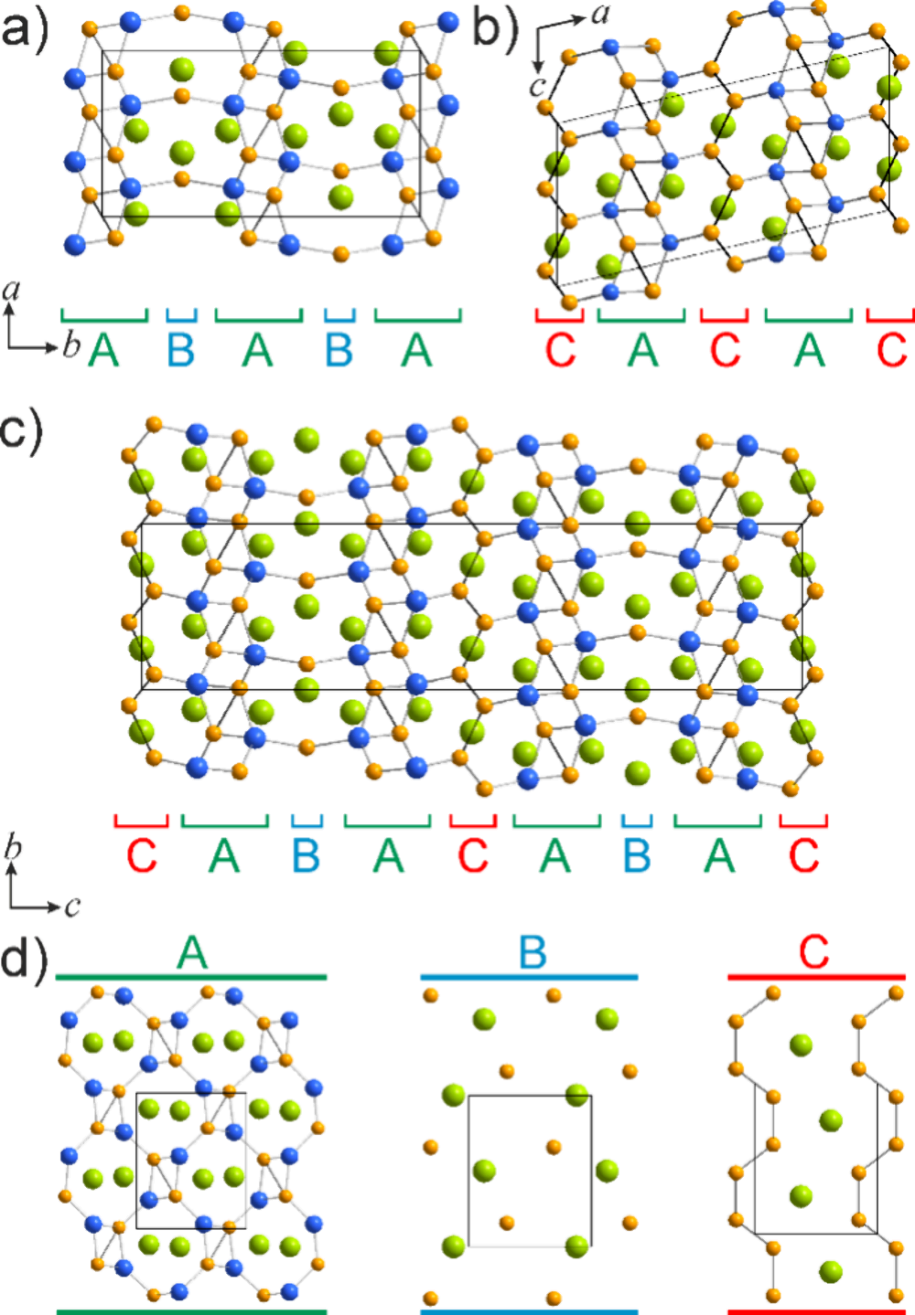
Comparison of the crystal structures of (a) La_3_Ni_3_Si_2_, (b) La_3_Ni_4_Si_2_, and (c) La_3_Ni_3.5_Si_2_ and (d) projections of the constituent slabs extending normal to the projection planes. La atoms are colored green, Ni atoms orange, and Si atoms blue.

Alternatively, the crystal structure of La_3_Ni_4_Si_2_ can be presented in terms of layered packing and is a part of the compositional sequence with a fixed La/Si ratio and close structural relationships. It is also worth noting that such sequences are rather common in the La–Ni–Si system with already reported two homologous series. This particular sequence here comprises La_3_Ni_3_Si_2_^17^ (Ce_3_Rh_3_Si_2_ structure type),^73^ La_3_Ni_3.5_Si_2_^17^ (Pr_6_Ni_7_Si_4_ structure type)^74^ and the newly uncovered La_3_Ni_4_Si_2_. Each of these structures consists of a set of alternating slabs, namely LaNiSi (A), LaNi (B) and LaNi_2_ (C) (Figure 6). The compositionally intermediate La_3_Ni_3.5_Si_2_ exhibits structural features of both La_3_Ni_3_Si_2_ and La_3_Ni_4_Si_2_. Slab A is the most complex being represented by a nearly planar rhombi-octagonal tiling. Such motifs of a different degree of corrugation are rather common in polar intermetallics, particularly with late transition metals.^75,76^ In the crystal structure of La_3_Ni_4_Si_2_, slabs C are represented by Ni dumbbell zigzag chains serving as connectors between the neighboring slabs A, slightly shifted along the *c* axis. Interestingly, interslab connectivity is established solely via Ni–Si contacts.

The coordination environment of the La positions is quite irregular due to large open faces with coordination numbers ranging from 12 to 13. La1 is surrounded by eight Ni, four Si and an additional La atoms, while La2 is coordinated to eight Ni and 4 Si. With some degree of approximation, the latter polyhedron can be described as an irregular equatorially bicapped pentagonal prism. Additionally, there are five and eight La atoms in their second coordination spheres, respectively, centering large, open faces. All Ni and Si positions are surrounded by capped trigonal La_6_ prisms. Both Ni positions are capped equatorially only with three (2Ni + Si) or four (Ni + 3Si) atoms, while the SiLa_6_ polyhedron is tricapped equatorially and monocapped axially solely by Ni atoms. Ni–Si and Ni–Ni contacts in the structure are concentrated in narrow ranges of 2.352–2.459(1) Å and 2.572–2.665(1) Å, respectively, at or slightly above the sum of the corresponding covalent radii.^14^ No Si–Si bonds could be observed in the structure.

As this composition stays away from three previous structures, it makes more sense to compare it to the other member of the homologous series we analyzed in our previous work. The total bonding spectrum is visibly dominated by the cation–anion contacts −106 + 46, while Ni–Ni bonding is practically absent–only 12 contacts and Ni–Si bonds are maximized −72 (Table S9). This picture is practically identical to two other members of the series, i.e. La_3_Ni_3_Si_2_ and La_3_Ni_3.5_Si_2_ as well as two other compositionally close compounds LaNi_2_Si and La_2_Ni_3_Si_2_.^17^ Of no surprise anymore, being not the major component quantitatively the heteroatomic Ni–Si contacts always provide the largest contributions to the total bonding. These contributions are comparable to the as a rule less represented homoatomic Ni–Ni bonds, while the cation–anion pairs contributions are minimal at the single bond level but statistically the most represented and therefore significant (15–30% of the total ICOHP).

*Note. At the time of submission, we were made aware of another recently published manuscript that reports the structural properties of La*_*3*_*Ni*_*4*_*Si*_*2*_*from powder data and provides a different perspective of this homologous series*.^37^

## Conclusions

The La–Ni–Si system appears to be a gold mine for compounds of compositional and structural diversity. The system was intensively explored with the first reports starting in 1965^77^ and the first attempt to construct an isothermal section at 670 K appeared 20 years later.^78^ Due to the enormous complexity, limitations of the available methods at that time and noncoordinated efforts later, the full picture of the system has not emerged until now. The recent known attempts to construct similar isothermal sections for the La–Ni–Si system at different temperatures cannot be considered reliable due to the significant number of missing known ternaries.^19,20^ The most recent attempt of a systematic phase space exploration in similar systems was carried out for Ce–Ni–Si, Gd–Ni–Si and Dy–Ni–Si in 2014–2016 reporting 26 and 20 and 21 compounds, respectively^36,79,80^ showing a noticeable difference between the light and heavy lanthanide systems. Despite numerous representations, the following research brought three more new compounds in Ce–Ni–Si,^37^ however, even more compounds exist there as was shown in this work.

**Figure 7:**
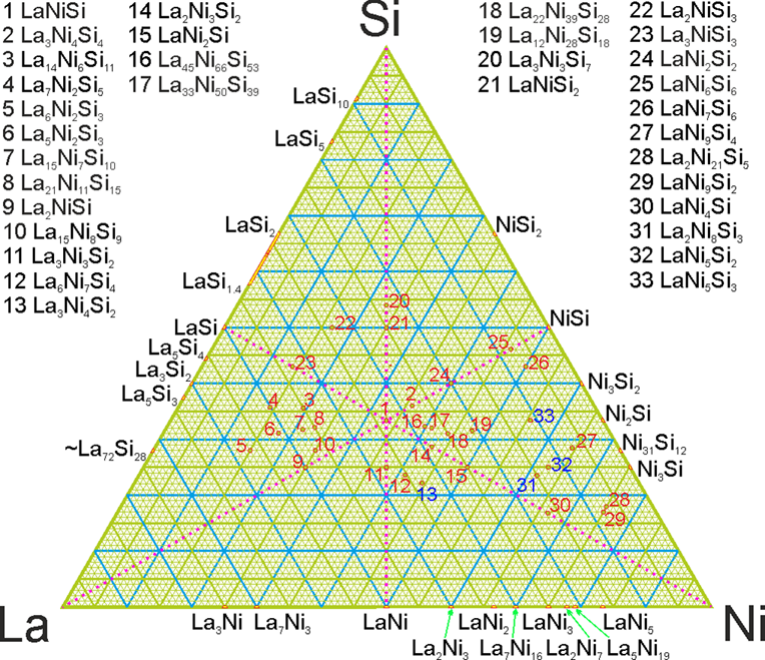
Map of the previously reported (marked with red numbers) and newly discovered (blue) La–Ni silicides.

Active exploration of the La–Ni–Si system are still ongoing. For instance, the area around “LaNi_6_Si_6_” has been examined together with isocompositional Ce–Ni–Si compounds,^36^ while the Si-poorer part in the middle of the system was explored in our previous work leading to eight new compounds within two homologous series.^16,17^ Certain experimental challenges are coming from significantly variable existence ranges for some compositionally close compounds. For example, La_3_Ni_4_Si_2_, in contrast to all other members of its homologous series, could not easily be detected at 1070 or even 970 K, while growing single crystals at 870 K required slow cooling and prolonged annealing. Shifting the exploration toward the Ni-richer areas revealed four more compounds, namely La_2_Ni_8_Si_3_, LaNi_5_Si_2_, LaNi_5_Si_3_ and La_3_Ni_4_Si_2_. LaNi_5_Si_2_ is the first Ni representative of a larger family typically formed by aurides, La_3_Ni_4_Si_2_ is a part of a local homologous series, while two remaining compounds bear some close analogies with phosphides or platinides. This brings the total number of discovered compounds to 33 as of now (Figure 7) and the ternary system in general as being one of the most populated, i.e. a gold mine of intermetallics. Taking into account the data from the closely related systems, even more compounds can be expected at least in the Ni-rich corner and still have to be discovered.

## Supporting Information Available

Rietveld refinements and additional details of the electronic structure calculations, Figures S1–S6, and Tables S1–S11 (PDF)
