# Supplementary material for: La–Ni–Si: A Gold Mine with a Diamond
Source: Inorg Chem. 2024 Nov 18;63(48):22761–70. doi: 10.1021/acs.inorgchem.4c03560 (PMC11615943; doi:10.1021/acs.inorgchem.4c03560)
Supplement: Supplementary file 1 — ic4c03560_si_001.pdf [file ic4c03560_si_001.pdf]

Supporting Information

La-Ni-Si: A Gold Mine with a Diamond

Volodymyr Smetana[1,2], Davide Grilli[2,3], Vitalii Shtender[4], Marcella Pani[,3], Pietro Manfrinetti*[,3], Anja-Verena Mudring*[,1,2,5]*

[1]*Intelligent Advanced Materials, Department of Biological and Chemical Engineering and iNANO, Aarhus University, 8000 Aarhus C, Denmark*
[2]*Department of Materials and Environmental Chemistry, Stockholm University, Stockholm 10691, Sweden.*
[3]*DCCI, Department of Chemistry and Industrial Chemistry, University of Genova, Genova I-16146, Italy; Institute SPIN-CNR, Genova I-16452, Italy*
[4]*Department of Chemistry – Ångström Laboratory, Uppsala University, Uppsala 75121; Sweden*
[5]*Department of Physics, Umeå University, Linnaeus väg 24, 901 87 Umeå, Sweden*

*Email: marcella.pani@unige.it; pietro.manfrinetti@unige.it; anja-verena.mudring@bce.au.dk; anja-verena.mudring@um.u.se;

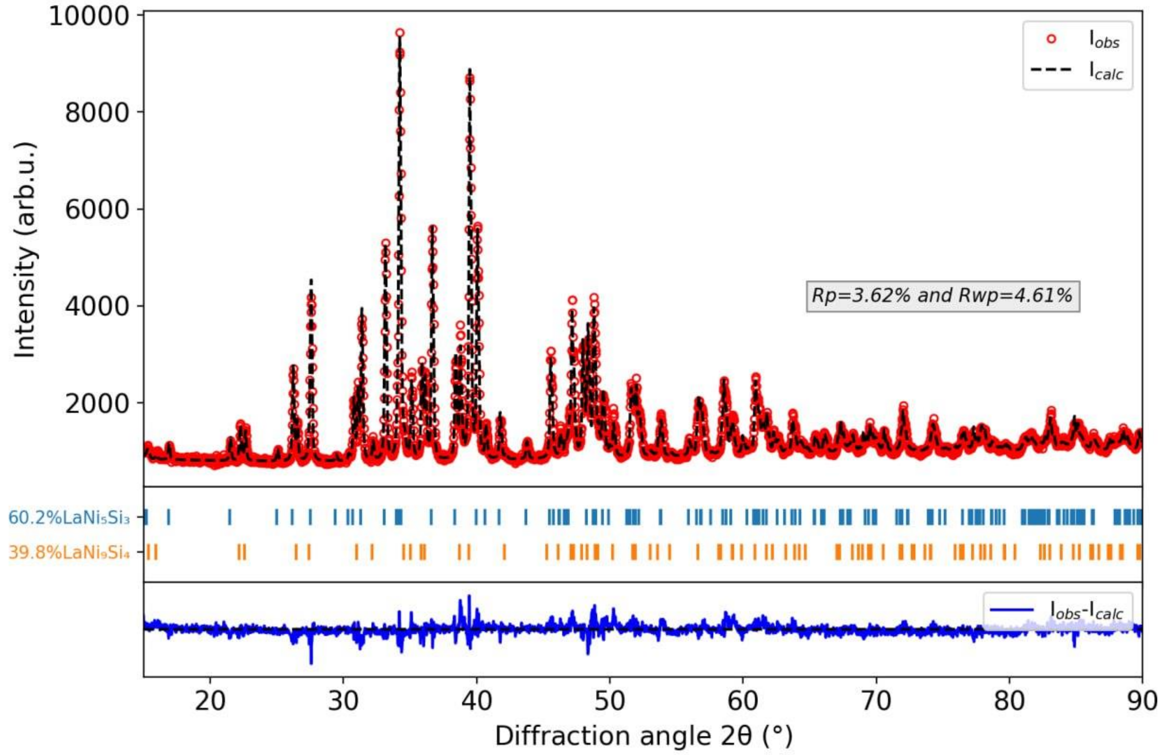

Figure S1. Rietveld refinement for a sample with nominal composition $La_{9.5}Ni_{57.2}Si_{33.3}$.

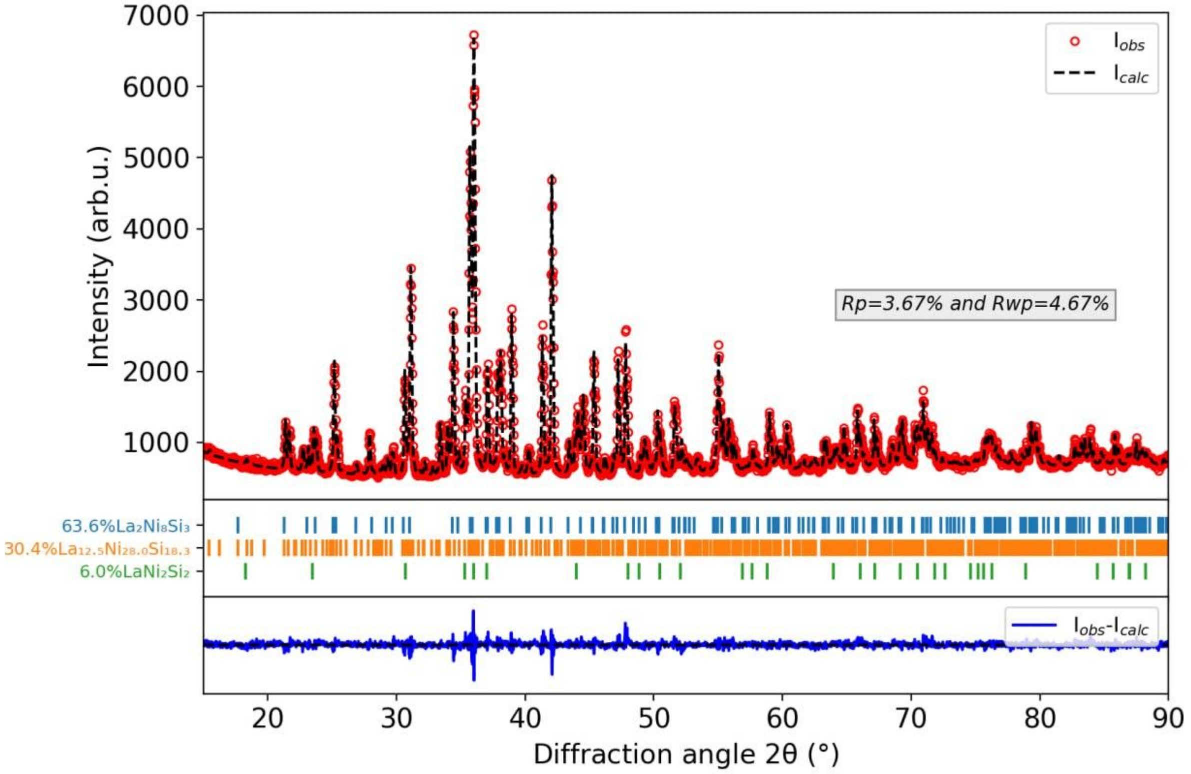

Figure S2. Rietveld refinement for a sample with nominal composition $La_{17.8}Ni_{54.6}Si_{27.6}$.

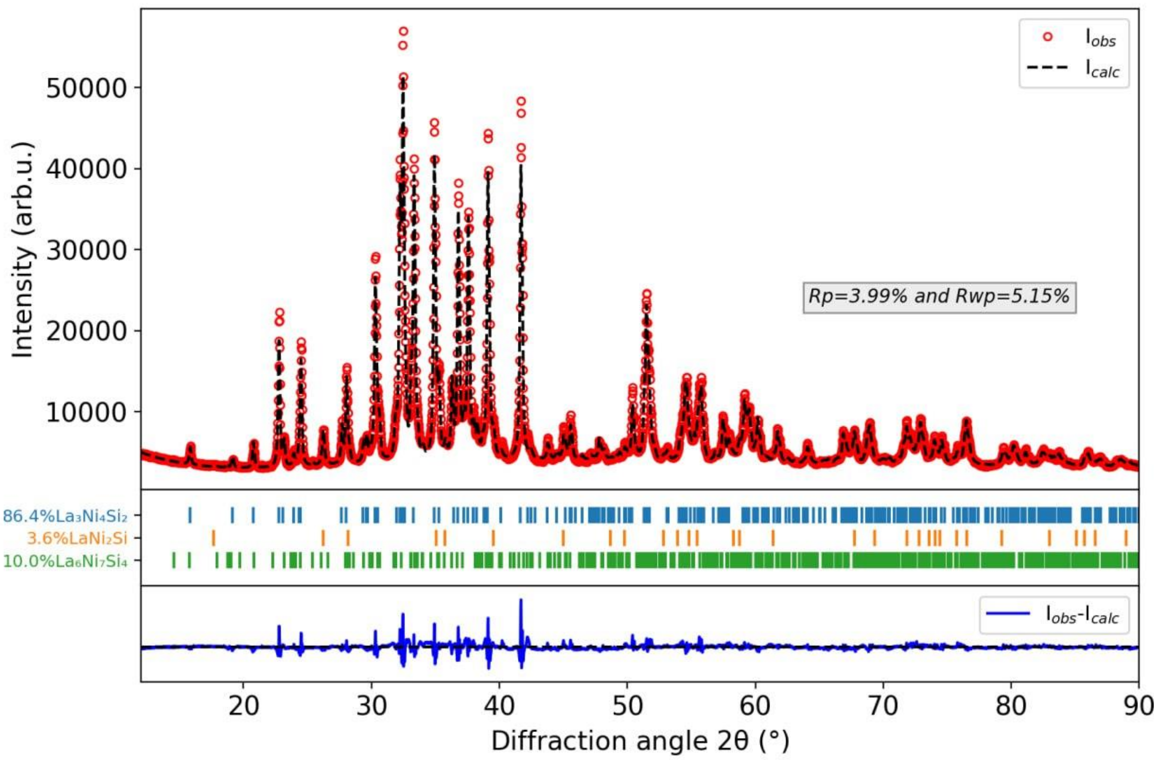

Figure S3. Rietveld refinement for a sample with nominal composition $La_3Ni_4Si_2$.

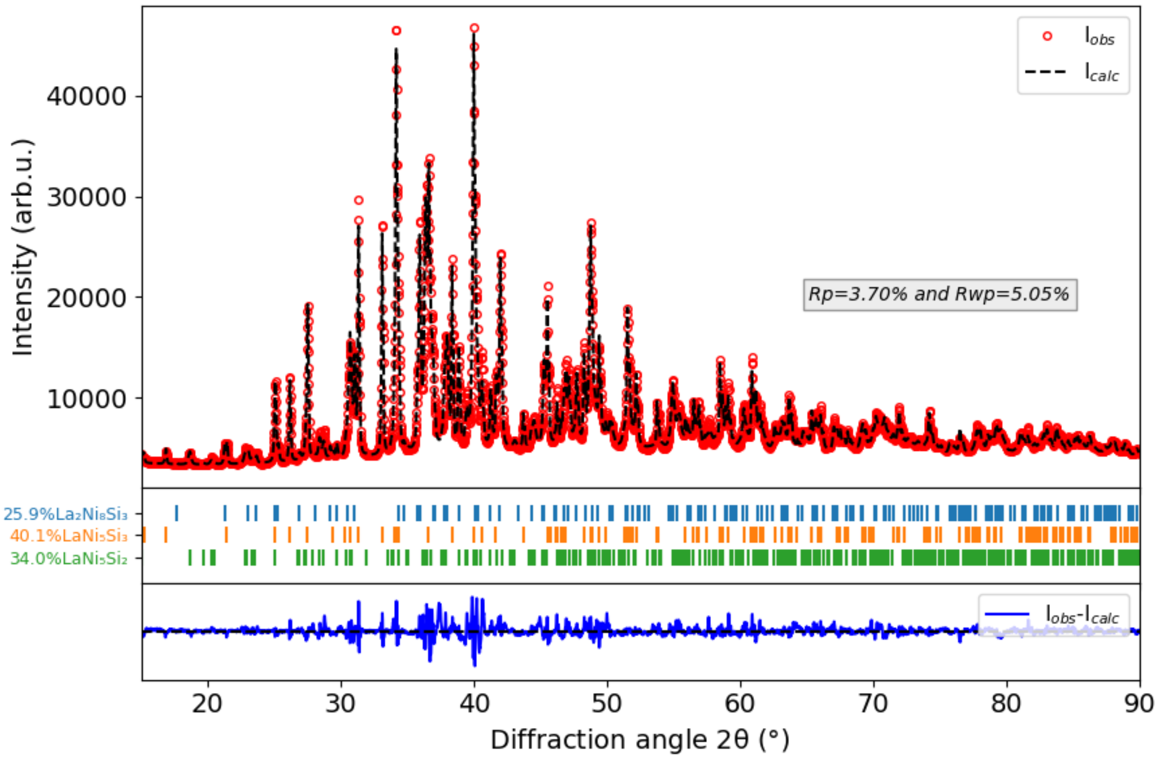

Figure S4. Rietveld refinement for a sample with nominal composition $La_{12.5}Ni_{58.75}Si_{28.75}$.

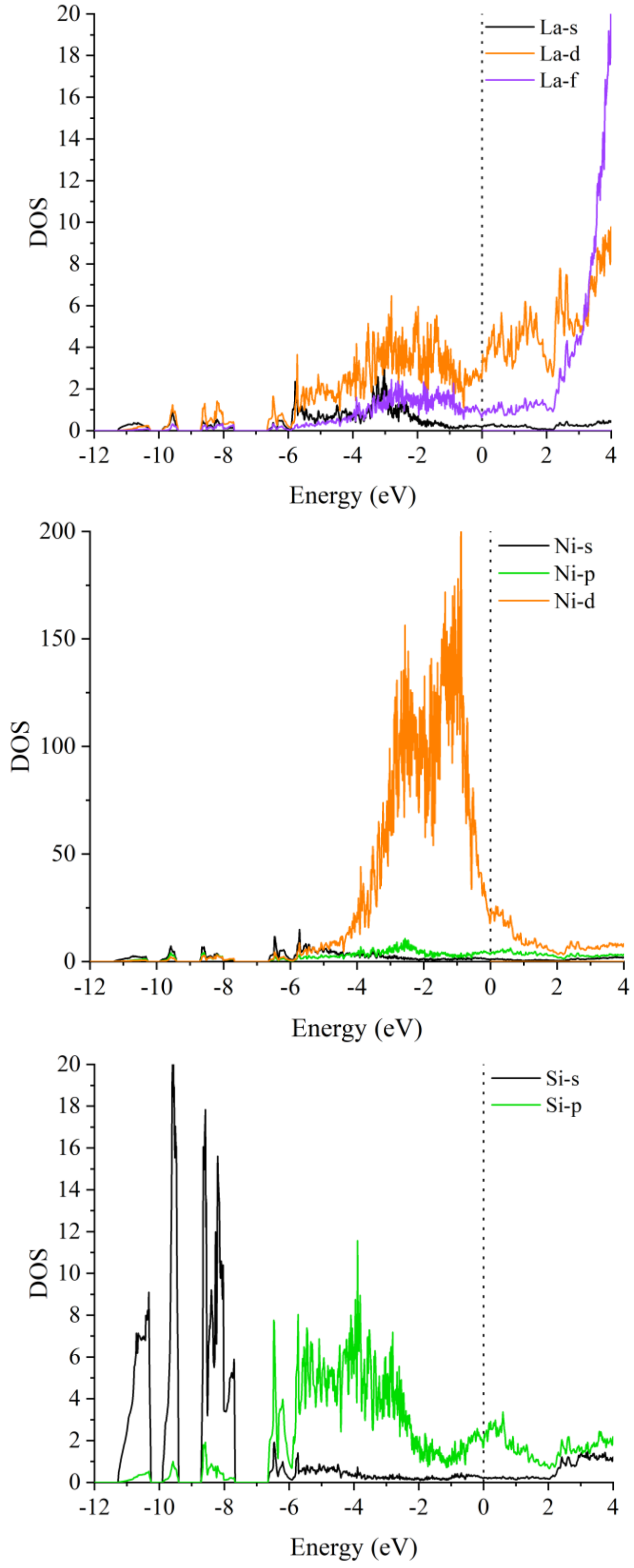

Figure S5. Element- and orbital-projected DOS for $LaNi_5Si_2$.

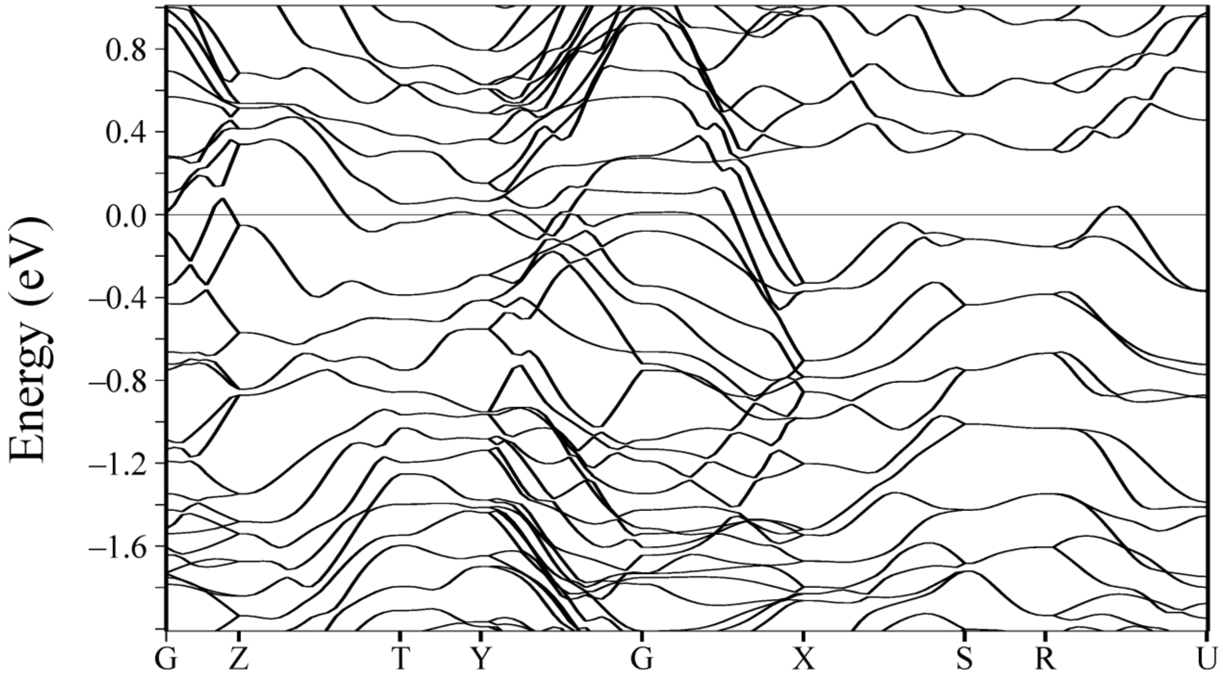

Figure S6. Band structure of $LaNi_5Si_2$ around E_F.

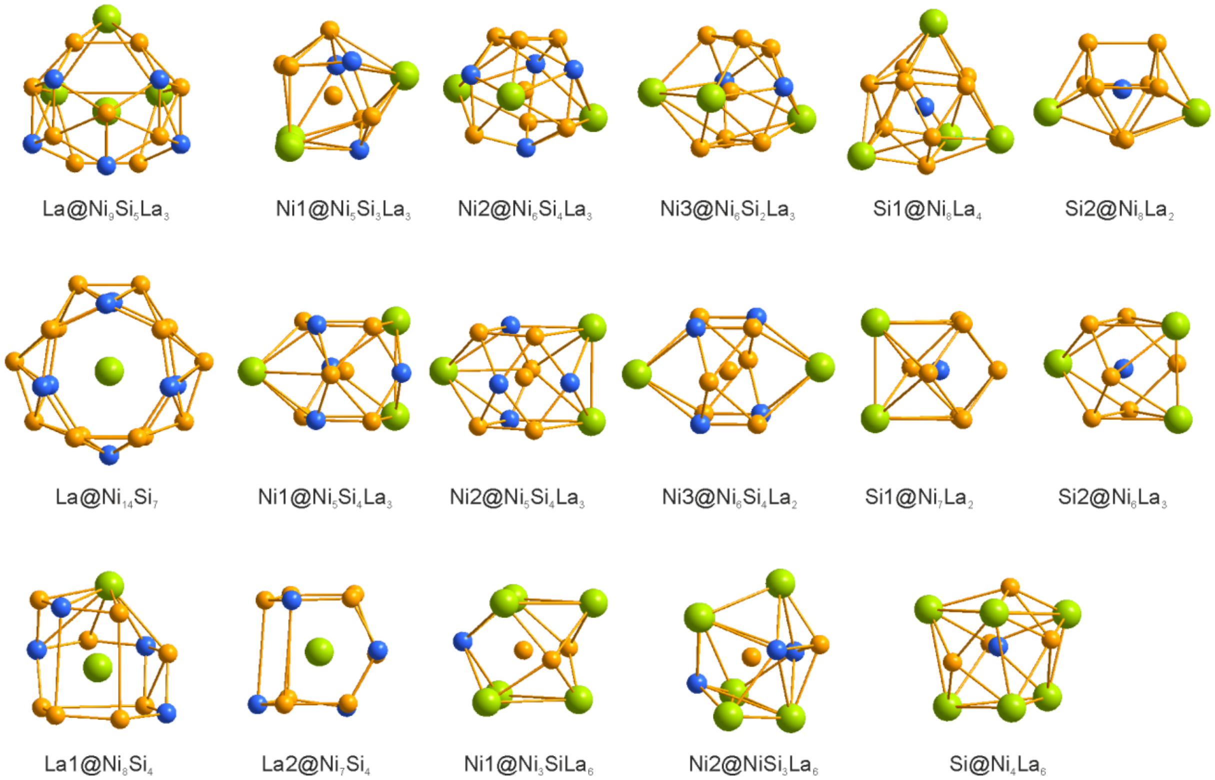

Figure S7. Coordination polyhedra of the atoms in the crystal structures of $La_2Ni_8Si_3$ (top row), $LaNi_5Si_3$ (middle row) and $La_3Ni_4Si_2$ (bottom row). La atoms are green, Ni – orange and Si – blue.

Table S1. Crystallographic data and details of structure refinements for $LaNi_5Si_2$, $CeNi_5Si_2$, $La_2Ni_8Si_3$, $LaNi_5Si_3$ and $La_3Ni_4Si_2$.

**Compound**	**$LaNi_5Si_2$**	**$La_2Ni_8Si_3$**	**$LaNi_5Si_3$**	**$La_3Ni_4Si_2$**	**$CeNi_5Si_2$**
**CCDC**	2375099	2375100	2375101	2375102	2375103
**Formula**	$LaNi_{4.98}Si_{2.02(1)}$	$La_2Ni_8Si_3$	$LaNi_5Si_3$	$La_3Ni_4Si_2$	$CeNi_{4.95}Si_{2.05(1)}$
**Formula weight, g·mol^{-1}**	487.94	831.77	516.73	707.75	488.10
**T (K)**	296(2)	302(2)	302(2)	296(2)	296(2)
**Crystal system**	Orthorhombic	Tetragonal	Orthorhombic	Monoclinic	Orthorhombic
SG	*Pnma*	$P4_2/nmc$	*Cmcm*	*C*2/*c*	*Pnma*
***a*, Å**	7.8223(7)	10.0278(3)	3.722(2)	15.819(1)	7.774(2)
***b*, Å**	6.3894(6)		11.759(5)	6.0068(5)	6.390(2)
***c*, Å**	17.843(2)	7.5047(4)	11.622(3)	7.4918(6)	17.539(5)
***β*, °**				103.163(5)	
***V*, Å^3**	891.8(2)	754.65(6)	508.7(3)	693.2(1)	871.2(4)
Z	8	4	4	4	8
**Density (g/cm^3)**	7.269	7.321	6.747	6.782	7.424
**μ (mm^{-1})**	30.297	30.897	26.876	28.910	31.510
***F*(000)**	1797	1520	956	1244	1802
**Index ranges**	$-11 \le h \le 11$	$-15 \le h \le 12$	$-5 \le h \le 4$	$-25 \le h \le 25$	$-10 \le h \le 10$
	$-8 \le k \le 8$	$-11 \le k \le 15$	$-14 \le k \le 15$	$-9 \le k \le 9$	$-8 \le k \le 8$
	$-25 \le l \le 23$	$-11 \le l \le 11$	$-15 \le l \le 14$	$-11 \le l \le 12$	$-23 \le l \le 24$
**Measured reflections**	17956	7670	2182	10378	7612
**Unique reflections**	1404	773	395	1487	1359
**Observed reflections**	1227	667	336	1285	1055
**Number of parameters**	89	37	31	43	88
**R_{int}**	0.0439	0.0621	0.0971	0.0397	0.0543
***R*, *wR* (observed)**	0.0296, 0.0617	0.0230, 0.0435	0.0488, 0.1141	0.0212, 0.0412	0.0238, 0.0537
**$\Delta\rho_{max}$ = (e·Å^{-3})**	1.43	1.10	2.05	1.98	1.30
**$\Delta\rho_{min}$ = (e·Å^{-3})**	–1.94	–1.26	–3.03	–1.27	–1.03

Table S2. Atomic Coordinates and Equivalent Isotropic Displacement Parameters for $LaNi_5Si_2$.

Site	*x*	*y*	z	U_{eq}	*SOF*
La1	0.22627(8)	0.75	0.68783(3)	0.0076(1)	1
La2	0.73827(8)	0.25	0.55897(4)	0.0084(1)	1
Ni1	0.3327(2)	0.25	0.49201(8)	0.0073(3)	1
Ni2	0.9001(1)	0.9453(2)	0.43968(5)	0.0080(2)	1
Ni3	0.8738(1)	0.9487(2)	0.66662(5)	0.0090(2)	1
Ni4	0.4423(1)	0.5471(2)	0.56779(6)	0.0087(2)	1
Ni5	0.5447(1)	0.0546(2)	0.70054(5)	0.0085(2)	1
Ni6	0.2947(2)	0.25	0.7533(1)	0.0082(5)	0.54(1)
Si6	0.2947(2)	0.25	0.7533(1)	0.0082(5)	0.46(1)
Ni7	0.0582(3)	0.25	0.6793(1)	0.0110(6)	0.41(1)
Si7	0.0582(3)	0.25	0.6793(1)	0.0110(6)	0.59(1)
Si1	0.6279(4)	0.75	0.6401(2)	0.0073(5)	1
Si2	0.3354(4)	0.25	0.6237(2)	0.0063(5)	1
Si3	0.0573(4)	0.25	0.4434(2)	0.0080(5)	1

Table S3. Atomic Coordinates and Equivalent Isotropic Displacement Parameters for $La_2Ni_8Si_3$.

Site	*x*	*y*	*z*	U_{eq}	*SOF*
La1	0.25	0.54238(3)	0.89958(4)	0.01059(9)	1
Ni1	0.04085(5)	0.12488(5)	0.03684(6)	0.0096(1)	1
Ni2	0.25	0.11384(7)	0.26215(9)	0.0098(1)	1
Ni3	0.25	0.62278(7)	0.35471(10)	0.0121(2)	1
Si1	0.4578(1)	0.5422(1)	0.25	0.0096(3)	1
Si2	0.25	0.25	0.0202(3)	0.0089(4)	1

Table S4. Atomic Coordinates and Equivalent Isotropic Displacement Parameters for $LaNi_5Si_3$.

Site	*x*	*y*	*z*	U_{eq}	*SOF*
La1	0	0.83536(9)	0.25	0.0112(4)	1
Ni1	0	0.19609(14)	0.06532(14)	0.0108(4)	1
Ni2	0	0.55145(14)	0.14549(14)	0.0113(5)	1
Ni3	0	0	0	0.0112(6)	1
Si1	0	0.3840(3)	0.0376(3)	0.0103(8)	1
Si2	0	0.1112(4)	0.25	0.0113(11)	1

Table S5. Atomic Coordinates and Equivalent Isotropic Displacement Parameters for $La_3Ni_4Si_2$.

Site	*x*	y	z	U_{eq}	*SOF*
La1	0.15386(2)	0.11107(4)	0.45712(3)	0.00881(6)	1
La2	0	0.62011(5)	0.25	0.00915(7)	1
Ni1	0.03602(3)	0.15332(9)	0.11143(7)	0.01166(10)	1
Ni2	0.28809(3)	0.15332(8)	0.15792(7)	0.00957(10)	1
Si1	0.16343(7)	0.38890(18)	0.11461(14)	0.00835(18)	1

Table S6. Atomic Coordinates and Equivalent Isotropic Displacement Parameters for $CeNi_5Si_2$.

Site	*x*	*y*	*z*	U_{eq}	*SOF*
Ce1	0.22521(5)	0.75	0.68850(2)	0.0096(1)	1
Ce2	0.73491(6)	0.25	0.56091(2)	0.0117(1)	1
Ni1	0.3320(1)	0.25	0.48980(5)	0.0098(2)	1
Ni2	0.89796(8)	0.9461(1)	0.43971(4)	0.0094(2)	1
Ni3	0.87409(9)	0.9473(1)	0.66595(4)	0.0115(2)	1
Ni4	0.43401(9)	0.5526(1)	0.56656(4)	0.0126(2)	1
Ni5	0.54153(9)	0.0529(1)	0.70145(4)	0.0104(2)	1
Ni6	0.2897(1)	0.25	0.75205(6)	0.0097(3)	0.818(8)
Si6	0.2897(1)	0.25	0.75205(6)	0.0097(3)	0.182(8)
Ni7	0.0472(2)	0.25	0.6775(1)	0.0097(5)	0.090(7)
Si7	0.0472(2)	0.25	0.6775(1)	0.0097(5)	0.910(7)
Si1	0.6236(3)	0.75	0.6423(1)	0.0098(4)	1
Si2	0.3367(3)	0.25	0.6237(1)	0.0061(4)	1
Si3	0.0516(3)	0.25	0.4442(1)	0.0097(4)	1

Table S7. Interatomic distances and bond statistics in $La_2Ni_8Si_3$.

		Distance (Å)	Contact/cell			Distance (Å)	Contact/cell
La1	La1	3.7027(4)	8	Ni2	Ni2	2.731(1)	4
La1	Ni1	2.8763(5)	16	Ni3	Ni3	2.393(1)	8
La1	Ni3	3.0598(6)	16	Ni3	Ni2	2.472(1)	8
La1	Ni1	3.0690(5)	16	Ni3	Ni1	2.4768(8)	16
La1	Ni1	3.1115(6)	16	Ni3	Ni3	2.552(1)	4
La1	Ni2	3.1397(7)	8	Ni3	Si1	2.369(1)	16
La1	Si2	3.0685(7)	8	Ni1	Si1	2.3165(9)	16
La1	Si1	3.250(1)	16	Ni1	Si2	2.4471(5)	12
La1	Si1	3.3574(7)	16	Ni1	Si1	2.4525(5)	16
Ni1	Ni1	2.5093(9)	8	Ni2	Si2	2.272(2)	8
Ni1	Ni1	2.6926(9)	8	Ni2	Si2	2.369(2)	8
Ni2	Ni1	2.5218(7)	16	Ni2	Si1	2.6075(5)	16
Ni2	Ni1	2.6962(6)	16				

Table S8. Interatomic distances and bond statistics in $LaNi_5Si_3$.

		Distance (Å)	Contact/cell			Distance (Å)	Contact/cell
La1	La1	3.722(2)	4	Ni3	Ni2	2.586(1)	16
La1	Si1	3.144(3)	16	Ni1	Ni2	2.688(2)	16
La1	Si2	3.227(4)	8	Ni1	Si1	2.233(4)	8
La1	Si2	3.244(6)	4	Ni1	Si1	2.404(2)	8
La1	Ni1	3.279(2)	16	Ni1	Si2	2.367(3)	8
La1	Ni2	3.376(2)	8	Ni2	Si1	2.259(4)	4
La1	Ni3	3.491(2)	8	Ni2	Si1	2.334(4)	8
La1	Ni2	3.553(2)	8	Ni2	Si2	2.331(2)	16
Ni1	Ni1	2.716(3)	4	Ni3	Si1	2.349(2)	16
Ni2	Ni2	2.429(3)	8	Si1	Si1	2.865(3)	4
Ni1	Ni3	2.428(2)	4				

Table S9. Interatomic distances and bond statistics in $La_3Ni_4Si_2$.

		Distance (Å)	Contact/cell			Distance (Å)	Contact/cell
La1	La1	3.4001(5)	4	La1	Si1	3.218(1)	14
La1	Ni2	2.7998(6)	8	La1	Si1	3.336(1)	8
La1	Ni1	2.8369(6)	8	La1	Si1	3.093(1)	8
La1	Ni1	2.8836(6)	8	La2	Si1	3.292(1)	8
La1	Ni1	2.9406(6)	8	La2	Si1	3.306(1)	8
La1	Ni2	3.0834(6)	8	Ni1	Ni1	2.571(1)	4
La1	Ni2	3.1556(6)	8	Ni1	Ni1	2.586(1)	4
La1	Ni2	3.4301(6)	8	Ni2	Ni2	2.665(1)	4
La2	Ni1	2.9675(6)	10	Ni2	Si1	2.328(1)	8
La2	Ni1	3.0877(6)	10	Ni2	Si1	2.353(1)	16
La2	Ni2	3.2711(6)	8	Ni2	Si1	2.389(1)	24
La2	Ni1	3.4540(7)	14	Ni1	Si1	2.458(1)	24
La1	Ni2	3.5434(6)	8				

Table S10. ICOHP data for all contacts in the crystal structure of $LaNi_5Si_2$

	Ni-Si triangles						Ni-Si inter												
length	2.272	2.334	2.353	2.322	2.375		2.387	2.334	2.484	2.474	2.318	2.301	2.4	2.416	2.497	2.484	2.452	2.304	2.35
no.	4	8	8	4	4		4	4	8	8	8	8	8	8	8	8	8	8	4
-ICOHP	2.34	1.94	1.9	2.19	1.95		1.76	2.1	1.51	1.66	2.29	2.25	1.82	1.9	1.45	1.65	1.76	2.56	1.88

	Ni-Ni 0y					Ni-Ni x0z							Ni-Ni inter						
length	2.49	2.59	2.53	2.49															
	8	3	9	6		2.652	2.583	2.805	2.729	2.75	2.68		2.506	2.461	2.476	2.522	2.483	2.432	
no.	4	4	4	4		4	8	8	8	4	8		8	8	8	8	8	8	
-ICOHP	1.05	0.91	0.89	1.03		0.97	0.97	0.69	0.85	0.82	0.85		1.14	1.39	1.31	1.16	1.3	1.43	

	La-Ni																		
length	3.39	3.368	3.15	3.092	3.378	3.198	2.998	2.966	2.918	3.443	3.169	3.126	3.02	3.153	3.113	3.059			
no.	4	8	8	8	4	8	8	8	8	8	8	8	8	8	8	8			
-ICOHP	0.27	0.32	0.47	0.39	0.27	0.36	0.43	0.5	0.39	0.24	0.32	0.31	0.3	0.32	0.28	0.26			

	La-Si											Si-Si			La-La				
length	3.611	3.573	3.356	3.297	3.236	3.499	3.458	3.255	3.226	3.168		2.878	2.385		4.412	4.497			
no.	8	8	4	4	4	8	8	4	4	4		4	4		4	4			
-ICOHP	0.16	0.19	0.29	0.35	0.46	0.23	0.25	0.37	0.4	0.41		0.49	2.2		0.06	0.05			

Table S11. Bond length, -ICOHP values and bonding contributions in $La_2Ni_8Si_3$, $LaNi_5Si_3$ and $La_3Ni_4Si_2$.

bond type	-ICOHP (eV/av.bond)	no/cell	-ICOHP (eV/cell)	contribution (%)
		$La_2Ni_8Si_3$		
La-La	0.17	8	1.36	0.44
La-Ni	0.41	72	29.5	9.48
La-Si	0.43	40	17.2	5.52
Ni-Ni	1.08	88	99.4	30.5
Ni-Si	1.83	92	161	54.1
		$LaNi_5Si_3$		
La-La	0.17	4	0.68	0.29
La-Ni	0.4	40	15.9	6.83
La-Si	0.63	28	17.7	7.59
Ni-Ni	1.01	48	48.7	20.9
Ni-Si	2.18	68	149	63.8
Si-Si	0.32	4	1.28	0.55
		$La_3Ni_4Si_2$		
La-La	0.19	4	0.76	0.37
La-Ni	0.44	106	46.5	22.7
La-Si	0.28	46	12.7	6.2
Ni-Ni	1.3	12	15.6	7.6
Ni-Si	1.79	72	129.2	63.1
